# Corticothalamic Synaptic Noise as a Mechanism for Selective Attention in Thalamic Neurons

**DOI:** 10.3389/fncir.2015.00080

**Published:** 2015-12-22

**Authors:** Sébastien Béhuret, Charlotte Deleuze, Thierry Bal

**Affiliations:** ^1^Unité de Neurosciences, Information et Complexité, Centre National de la Recherche Scientifique FRE-3693Gif-sur-Yvette, France; ^2^Institut National de la Santé et de la Recherche Médicale U 1127, Centre National de la Recherche Scientifique UMR 7225, Sorbonne Universités, UPMC Univ Paris 06 UMR S 1127, Institut du Cerveau et de la Moelle ÉpinièreParis, France

**Keywords:** thalamic gateway, thalamocortical system, sensory transfer, selective attention, corticothalamic feedback, synaptic noise, gain control, activity decorrelation

## Abstract

A reason why the thalamus is more than a passive gateway for sensory signals is that two-third of the synapses of thalamocortical neurons are directly or indirectly related to the activity of corticothalamic axons. While the responses of thalamocortical neurons evoked by sensory stimuli are well characterized, with ON- and OFF-center receptive field structures, the prevalence of synaptic noise resulting from neocortical feedback in intracellularly recorded thalamocortical neurons *in vivo* has attracted little attention. However, *in vitro* and modeling experiments point to its critical role for the integration of sensory signals. Here we combine our recent findings in a unified framework suggesting the hypothesis that corticothalamic synaptic activity is adapted to modulate the transfer efficiency of thalamocortical neurons during selective attention at three different levels: First, on ionic channels by interacting with intrinsic membrane properties, second at the neuron level by impacting on the input-output gain, and third even more effectively at the cell assembly level by boosting the information transfer of sensory features encoded in thalamic subnetworks. This top-down population control is achieved by tuning the correlations in subthreshold membrane potential fluctuations and is adapted to modulate the transfer of sensory features encoded by assemblies of thalamocortical relay neurons. We thus propose that cortically-controlled (de-)correlation of subthreshold noise is an efficient and swift dynamic mechanism for selective attention in the thalamus.

## Introduction

### Importance of the corticothalamic feedback

Nearly all sensory information transmitted to the neocortex, and thus critical to perception and attention, is relayed by the thalamus. Thalamocortical (TC) neurons in the dorsolateral geniculate nucleus (dLGN) relay visual input from retinal ganglion cells to the cortex, from which they receive massive feedback (Erişir et al., 1997; Van Horn et al., 2000), as well as synaptic and non-synaptic influences from other sources (Casagrande et al., 2005). A third of the synapses received by a thalamocortical cell have a direct cortical origin. Disynaptic inhibitory inputs from GABAergic local interneurons and neurons of the reticular thalamic nucleus (NRT), which both receive monosynaptic cortical inputs, account for another third of synapses onto TC cells (Erişir et al., 1997; Van Horn et al., 2000; Sherman and Guillery, 2002). Thus, two-third of synapses contacting a single TC neuron are related directly or indirectly to the activity of layer 6 corticothalamic (CT) axons. The CT feedback has long been considered as having a strong influence on the control of sensory information transfer by thalamocortical cells (Sherman and Koch, 1986; Koch, 1987; Ahissar, 1997; Sherman, 2001; Sillito and Jones, 2002) and could be involved in selective attention (O'Connor et al., 2002; Casagrande et al., 2005; Saalmann and Kastner, 2009), with recent evidences pointing out that corticothalamic feedback alters orientation-selectivity in human LGN during attention (Ling et al., 2015) and is involved in goal-directed attention in the mouse, via the disynaptic pathway involving the NRT (Ahrens et al., 2014). However, the mechanisms of cortically-driven attention in the thalamus remain an intriguing open question.

A well-known hypothesis endows the corticothalamic feedback and the NRT with a searchlight function (Crick, 1984) or focal attention (Montero, 1999) by enhancing selectively the receptivity of targeted TC neuron populations to attended sensory features. Others consider the thalamus as an “active blackboard” onto which the cortex could write down the results of its computation (Mumford, 1991). Some of these exciting hypotheses only began to be demonstrated recently (Wimmer et al., 2015) in awake and attentive animals due to technical limitations using conventional *in vivo* approaches.

Studies of the functional impact of the CT feedback *in vivo* are mainly restricted to extracellular recordings. They reveal an increased contrast gain in the anesthetized macaque (Przybyszewski et al., 2000), a spatial sharpening of thalamic receptive field and its ON-OFF antagonism (Temereanca and Simons, 2004), the facilitation of lateral geniculate nucleus activity in the awake cat (Waleszczyk et al., 2005), or attentive monkey (McAlonan et al., 2006), the synchronizing action on thalamic neurons involved in the detection of co-aligned elements in the visual field (Murphy et al., 1999; Wang et al., 2006) as well as the enhancement of the surround antagonism during motion processing (Sillito et al., 2006).

Synaptic activity originating from projections of cortical layer 6 is not well known, with a substantial proportion of corticothalamic cells that remain weakly active or silent in anesthetized or awake animals. Nevertheless, there are behavioral circumstances in which the corticothalamic feedback could be engaged (see Discussion), thus providing TC neurons with synaptic activity of cortical origin.

### The corticothalamic feedback provides neurons with synaptic noise

*In vivo*, neurons are constantly exposed to background barrages of synaptic inputs, called “synaptic noise,” which likely interact with their membrane properties (Steriade, 2001; Destexhe et al., 2003) and impact on their response to “meaningful” sensory synaptic input (Destexhe and Paré, 1999; Anderson et al., 2000). The sensitivity of individual TC cells to sensory input can be assessed by their input-output transfer function, which evaluates the probability of firing in response to synaptic inputs of given amplitudes. In cortical cells the fluctuation of membrane conductances is able to change the gain, i.e., the slope of the input-output spike transfer function (Hô and Destexhe, 2000; Chance et al., 2002; Shu et al., 2003), and to increase neuronal responsiveness (Hô and Destexhe, 2000). Likewise, thalamocortical cells recorded *in vivo* are also in a high-conductance state, in particular during corticothalamic barrages (Contreras et al., 1996; Steriade, 2001), enabling interactions with the synaptic input during sensory integration.

The increase in detection sensitivity to sensory input by TC neurons likely results from a change in the tuning of cortically-driven synaptic inputs during attention. In the higher level visual cortical area V4, individual neurons respond to attended stimuli that are not salient enough to elicit a response when unattended. The lower threshold of response and increase in sensitivity is reflected in a leftward shift in the contrast-response function without a substantial increase in the firing response to high-contrast stimuli (Reynolds et al., 2000). During a similar attentive task, the firing of thalamic relay neurons increases slightly (Casagrande et al., 2005; McAlonan et al., 2006, 2008). However, individual TC neurons were recorded extracellularly, providing no information on the intracellular mechanisms responsible for the attention-dependent effects.

The precise structure of thalamic activity resulting from cortical modulation and the nature and origin of the CT feedback are still elusive. To date it remains unclear how the cortical input affects the transfer of sensory signals to the neocortex, and the dynamical brain processes that are involved in the control of thalamic activity. A detailed modeling of activity of layer 6 seems presently an unreachable target, since it would require taking into account network interactions with all other cortical layers and other related cortical areas. Instead we model the synaptic noise as a configurable activity pattern transmitted by fluctuating excitatory and inhibitory conductances for which we control the statistical structure. We investigate how the cortical input affect the relay of sensory signals by injecting these patterns into biological TC cells in visual and somatosensory thalamic nuclei.

### Synaptic noise acts at three different levels within the thalamocortical circuit

In a series of studies using dynamic clamp in slices *in vitro*, we show that synaptic noise acting at the ionic channel, neuron and network levels, tunes the relay function of the thalamus, thus promoting the idea that the thalamus is more than a passive gateway for the relay of sensory inputs to the neocortex.

The ionic channel level: In TC cells, when membrane potential is depolarized, the T-type calcium channels were previously thought to be inactivated. We found that the rapid membrane potential fluctuations that result from background synaptic noise influence the dynamics of T-type channels, making a fraction of these channels available for activation, thus boosting the spike responsiveness of individual thalamocortical neurons during wake-like states (Deleuze et al., 2012). We demonstrate that the activation of T-channels during wake-like states is a major determinant for single-spike and burst occurrence during tonic firing, forming a multi-spike code that improves coding capabilities, and provides robustness to the thalamocortical transfer of sensory inputs.

The neuron level: By adjusting synaptic conductance parameters, such as the amplitude of fluctuations or the ratio of excitation and inhibition, we demonstrate that synaptic noise tunes the gain and sensitivity of individual neuron spiking probability function (Wolfart et al., 2005), which results from the stochastic resonance between sensory synaptic inputs and membrane potential fluctuations. By regulating the intensity of background activity, the cortex could thus exert a fast and efficient control of the thalamic relay through instantaneous adjustment of gain and of bursting probability, which may be related to selective attention mechanisms.

The network level: A novel property, which could participate to the cellular mechanisms of selective attention, emerges at the level of the neuronal population, where recipient cortical cells receive input from a number of thalamocortical cells. The stochastic resonance distributed in the afferent network of thalamic neurons provides an emerging signal filtering property critically controlled by synaptic noise (de-)correlation across the thalamic assembly. We found that synaptic noise, presumably under the fast control of neocortical feedback, facilitates the synchronization of spikes propagated to the neocortex by decorrelating the activity of thalamocortical cells (Béhuret et al., 2013). The resulting population-based stochastic facilitation can selectively boost the information transfer of sensory signals to neocortex.

We therefore propose that corticothalamic feedback exerts its function not only by exciting or inhibiting thalamocortical cells, but also by using a separate “channel” of modulatory information: The statistical structure of the cortical background synaptic input, including the mean and variance of synaptic conductances, the ratio of excitation and inhibition, and the correlation of synaptic noise across TC cells. From these works emerges the hypothesis that cortically-induced synaptic noise endows TC neurons with a function of selective attention, where rapid modulations of the statistical structure of synaptic noise determine the selection and deselection of sensory signals during attention.

### The hypothesis: synaptic noise provides a mechanism for selective attention in thalamic neurons

Attentional modulation originating in higher-level visual areas and focusing its action on low-level visual areas is central to the “Reverse Hierarchy Theory” (Hochstein and Ahissar, 2002). It posits that the “pop-out” phenomenon, which allows one to perceive a visual stimulus without being aware of the smaller details that it is made of, emerges initially from activity within high-level areas using their large receptive fields. Filling-in perceptual details demands focused attention, and it is proposed that later “reentrant” feedback to lower levels progressively adds details available in the small receptive fields found in primary areas. The nature of this feedback is unknown and we speculate that synaptic bombardment directed to neurons encoding specific features in the thalamocortical system could play a role in the selection of relevant sensory signals.

A number of experimental observations indicate that correlations present in thalamic and cortical activities change during focused attention. On the one hand, some studies report increased correlations in the alpha-beta (8–30 Hz) (Bekisz and Wróbel, 1993, 2003) and gamma (30–80 Hz) (Bouyer et al., 1981; Fries et al., 2001; Fries, 2009) bands. On the other hand, studies report decreased correlations in LFP signals during attentive tasks (Cohen and Maunsell, 2009; Mitchell et al., 2009), including in the alpha-beta bands (Fries et al., 2001). It is possible that changes in activity correlation depend on the attentional goals, as it is suggested by functional magnetic resonance imaging data recorded from human visual cortex (Al-Aidroos et al., 2012).

In this paper we propose a mechanism to reconcile these opposing findings: It was stated in Sillito et al. (1994) that “Selective attention may involve the cortical feedback to focus the appropriate circuitry onto the attended stimulus feature.” We suggest that this focus might be achieved—by decreasing correlations—, improving sensory coding of selected stimulus features, while the surrounding subnetworks coding for other features irrelevant to the task, would relax in a state of correlations associated with lower sensitivity. Therefore, we propose that (de-)correlation of the synaptic bombardment across TC neurons at the network level provides a plausible mechanism for selective attention in the thalamocortical system.

## Synaptic bombardment tunes spike transfer to cortex in individual thalamic neurons

### Synaptic noise controls the cell's sensitivity to synaptic inputs

**Figure 1:**
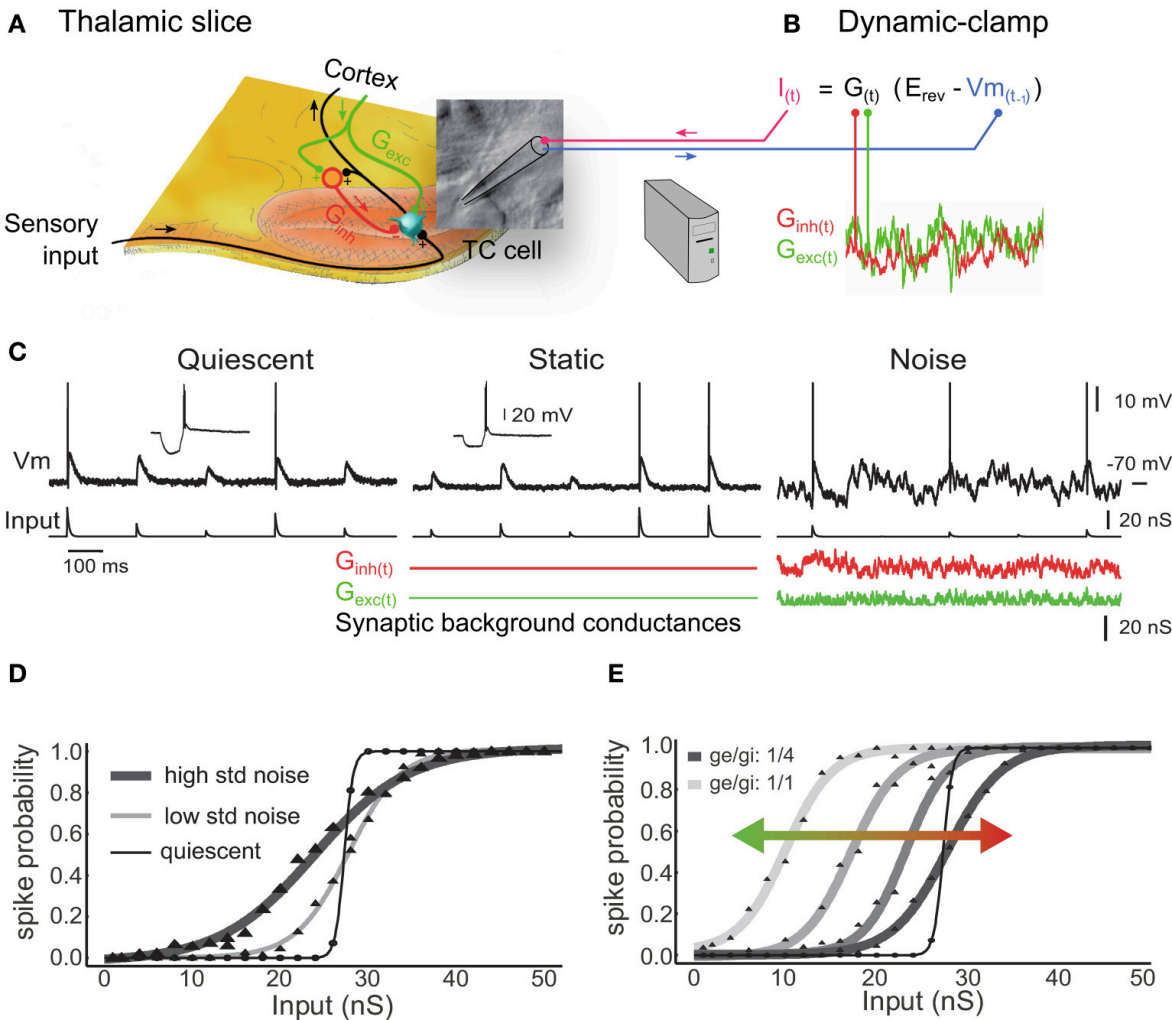
**Synaptic noise tunes the transfer function of thalamocortical cells recorded *in vitro***. **(A)** Illustration of a thalamic slice where a TC cell is recorded using a patch pipette. **(B)** The dynamic-clamp technique is used to stimulate TC cells with artificial sensory inputs and synaptic conductances, thus mimicking the impact of the corticothalamic feedback during sensory integration. **(C)** Voltage during injection of discrete retinal-like input conductance (quiescent) and with additional inhibitory plus excitatory background conductance that were either non-fluctuating (static) or stochastically fluctuating (noise). Combined inhibitory and excitatory conductances reduced the input resistance to ~50% (insets in quiescent and static). **(D)** Probabilities of input conductance strengths to evoke at least one spike within a 20 ms delay, fitted to sigmoid functions. In the noise condition (but not static; not shown, see in Wolfart et al., 2005), a multiplicative gain is induced, corresponding to a slope change of the response curve, and characterized by an increased sensitivity to small inputs and a decreased sensitivity to large inputs. Decreasing the variance of noise conductance values from high voltage variance noise (high std noise; 3.65 mV; *n* = 24) to low noise (low std noise; 2.6 mV; *n* = 5) changes the input-output slope and the sensitivity to small inputs. **(E)** Changing the ratio of excitatory/inhibitory conductances (1/1, 1/2, 1/3, and 1/4) induces an additive gain that shifts the dynamic input sensitivity range of the transfer function toward smaller inputs (leftward green shift) for higher ratios and toward larger inputs for lower ratios (rightward red shift). Modified from Wolfart et al. (2005).

In this section we illustrate how background synaptic noise affects individual thalamic neurons. We used the dynamic-clamp technique to reproduce the electrical impacts of the opening of ion channels in the membrane of intracellularly recorded biological TC neurons by injecting artificial conductances at the recording site through the glass pipette (Figure 1A). Dynamic-clamp relies on establishing a real-time loop between the computer-controlled injected current and the constantly updated and recorded membrane potential (Figure 1B, reviewed in Piwkowska et al., 2009). We simulated the noisy synaptic environment of the activated “wake-like” state in LGN and somatosensory thalamic neurons recorded *in vitro*. We injected sequences of sensory-like AMPA conductances (Figure 1C, quiescent) together with thousands of static (Figure 1C, static) or fluctuating (Figure 1C, noise) cortical synaptic inputs, mimicked by excitatory and inhibitory synaptic background conductances. In those protocols the sensory AMPA conductance amplitudes are randomly generated to reproduce a realistic spectrum of input synchrony degree. We describe below that background conductance noise significantly changes the burstiness and the input-output transfer function of thalamic relay neurons (Wolfart et al., 2005).

The probabilistic input-output curve in Figures 1D,E defines the neuronal responsiveness over a range of inputs of different amplitude and are characterized by their slope and position, forming multiplicative and additive gains, respectively (Rothman et al., 2009; Silver, 2010) (this is similar to the psychometric curves of contrast-response functions when probing the correlates of attention in monkey neurons).

We found that a step-like transfer function characterized the response of TC neurons in absence of subthreshold membrane fluctuations (quiescent condition in Figures 1C,D), providing a steep probabilistic input-output curve that presents poor encoding capabilities. Conversely, under the influence of noise the response probability was linearized, adopting intermediate values between 0 and 1 over a larger dynamic input range (high and low std noise in Figure 1D). This feature provides great flexibility for a possible top-down control of the thalamocortical transfer function through at least two mechanisms detailed in the following.

First, changing the variance of background conductance, which is reflected by a change in amplitude of voltage fluctuations, tunes the input-output gain of TC cells and their sensitivity to sensory inputs (Figure 1D). For instance, an EPSP generated by a 20 nS conductance is not detected in the quiescent state, but becomes progressively detectable when increasing the amplitude of stochastic membrane potential fluctuations. This multiplicative scaling by noise, which corresponds to a change in the slope of the transfer function, can be explained by the fact that the probability for small-amplitude inputs to evoke a spike can only be enhanced by noise (floor effect), whereas for larger-amplitude inputs, the probability can only be reduced by noise (ceiling effect) (Shu et al., 2003). This effect is linked to stochastic resonance, where synaptic noise-induced fluctuations randomly adding up to sensory-evoked EPSPs produce a linearized input-output transfer function. Note that the stochastic resonance effect is also of importance for information transfer at the population level and will be described later.

Second, changing the ratio of conductance excitation to inhibition shifts the response curve along the input axis. Figure 1E shows that decreasing the strength of the inhibitory conductance results in a leftward shift of the curve and an increase in the cell's sensitivity to smaller sensory EPSPs (green arrow). Conversely, increasing the strength of the inhibition results in a rightward shift of the curve and an increase in the cell's sensitivity to larger sensory EPSPs (red arrow). We tested several ratios of excitatory to inhibitory conductance, ranging from 1/1 to 1/4, that produced only little changes in membrane potential (Figure 1E, Vm mean ± SD; 1/1 ratio: −64.8±1.0; 1/2 ratio: −66.2 ± 1.1; 1/3 ratio: −66.9±1.2; 1/4 ratio: −66.2±1.3; *n* = 4), but were associated with significant shifts of the response curve (1/4 ratio: 53.5 ± 10.8 nS versus 1/1 ratio: 37.6 ± 10.3 nS; *p* = 0.034; one-sided test; *n* = 4).

In conclusion we show here two separate mechanisms by which background synaptic activity is able to control the input-output curve of TC neurons in a flexible manner, by changing either the gain (slope change = multiplicative change) or the sensitivity (position on the x-axis = additive change) of the neuronal transfer function or both. Consistently with the effects of synaptic noise that were observed in cortical neurons (Shu et al., 2003), we propose that the variance of synaptic noise amplitude and the ratio of conductance excitation to inhibition in thalamic neurons is a potent dual mechanism for sensory transfer modulation, which is separate from the classical Vm modulation that occurs through discrete postsynaptic excitation and inhibition. However, it remains unclear whether or not the cortex is capable of adjusting these parameters precisely and rapidly. These mechanisms remain a possibility that should be tested experimentally, for example by recording thalamic neurons intracellularly to assess individual conductance fluctuations and conductance ratios during increasingly-demanding attentive tasks.

### Interactions of subthreshold synaptic fluctuations with the T-type calcium current

**Figure 2:**
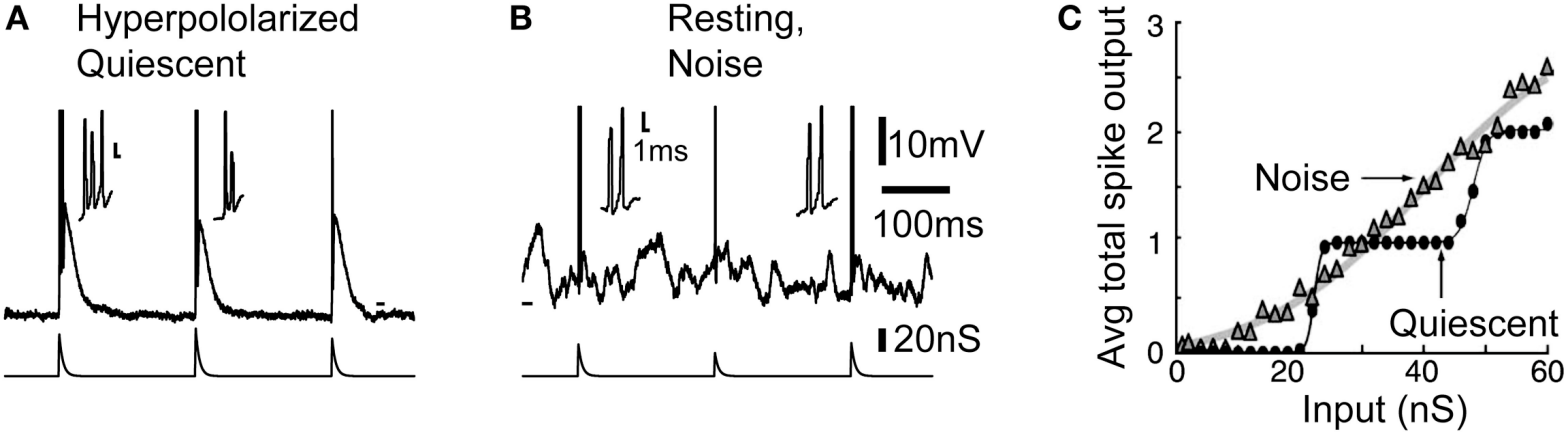
**Noise linearizes the transfer function of thalamocortical neurons through increased burstiness**. **(A)** Typical burst response in the quiescent hyperpolarized state. **(B)** Bursts also occurs in response to inputs when the neuron is depolarized by background synaptic noise. **(C)** During resting condition, plotting the average total number of spikes per burst response against the input shows that noise linearized the staircase-like transfer function across the whole input range. Modified from Wolfart et al. (2005).

A striking difference with cortical pyramidal neurons is that in quiescent thalamocortical cells, the gain is highly dependent on membrane potential level and input frequencies. In TC cells low-threshold calcium current boosts the response to synaptic inputs at hyperpolarized levels, for low frequencies limited to approximately < 10 Hz (McCormick and Feeser, 1990) (Figure 2A). This effect is explained by a cumulative inactivation of the T-type channels at higher frequencies. The inability of T-type channels to follow high frequencies provides thalamocortical cells with low-pass filter properties when they are hyperpolarized.

**Figure 3:**
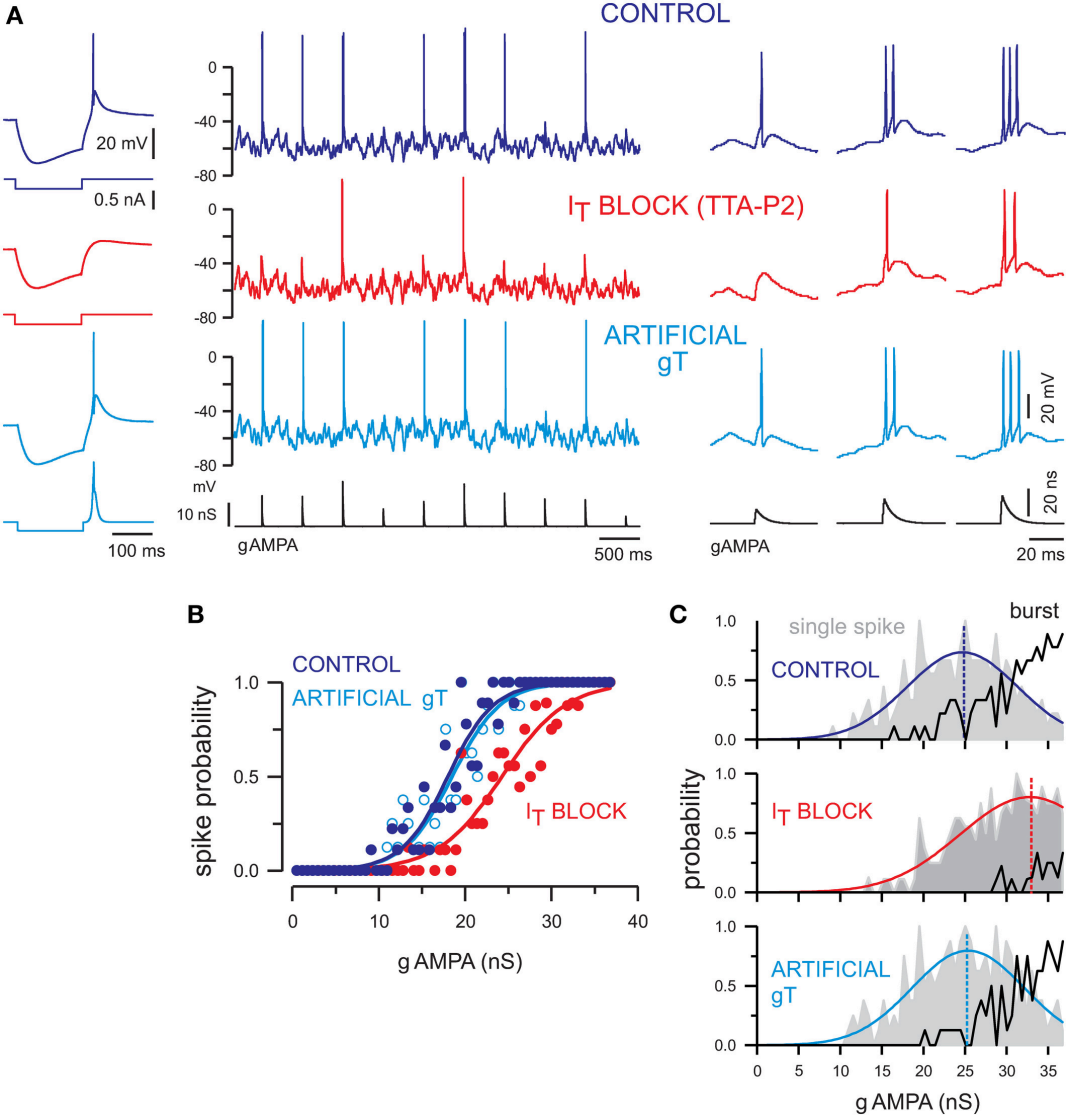
**T-current tunes the transfer function of TC neurons and contributes to single-spike and burst firing at depolarized membrane potentials**. **(A)** Left panel. The rebound low-threshold spike evoked in a TC neuron following a hyperpolarizing current step (control condition) is blocked by TTA-P2 and restored by dynamic clamp injection of gT. Middle panel. Voltage traces of a TC neuron injected with a sequence of AMPA conductances (gAMPA) of different amplitudes in control condition and in the presence of TTA-P2. The neuron received the same fluctuating excitatory and inhibitory conductance noise in each condition and displayed a mean membrane potential of −58 mV. The smallest AMPA conductances failed to evoke a spike when the T-current was blocked (red) and the spike probability was restored upon artificial gT injection (light blue). Right panel. Zoom on the firing activity in response to gAMPA of increasing amplitudes from the recording shown on the middle panel. Both single-spike and burst responses were conditioned by the presence of the T-current. **(B)** Transfer functions of the neuron presented in **(A)** show that the T-current block shifted the input-output curve toward larger AMPA conductances [same color code as in **(A)**]. Recovery was obtained with injection of gT. **(C)** Histograms present the probability of single-spike (gray area) and burst (black curve) generation as a function of the gAMPA amplitude in each condition. In the absence of T-current, the single-spike probability curve was shifted toward larger gAMPA and the burst probability was drastically reduced. The single-spike probability was fitted to a Gaussian function (colored line) to estimate the gAMPA conductance leading to the maximal probability (dashed line). Modified from Deleuze et al. (2012).

However, in presence of noise, this voltage-dependent response behavior is largely reduced, and the gain of the input-output curve characterizing the response probability remains similar at the potentials and frequencies tested (for details see Figures 3, 4 in Wolfart et al., 2005). Therefore, the presence of subthreshold voltage fluctuations masks the intrinsic, non-linear response behavior of thalamocortical cells, and equips them with a robust, quasi voltage-independent transfer function (Wolfart et al., 2005; Deleuze et al., 2012).

We found that part of this property of linearization results from increased burstiness. In the noise condition, even at resting and depolarized potentials where T-type calcium channels are thought to be fully inactivated, high-frequency multi-spike responses, made of two or three and sometime four spikes, often occurred, therefore mixing single-spikes and short duration bursts in response to inputs (Figure 2B). Without synaptic background, the cell behaves as a high-pass filter, detecting only strong inputs with no discrimination of strength above a certain threshold (see the staircase-like response in Figure 2C, quiescent). Conversely, in presence of noise the number of spikes grows proportionally to input strength on average, resulting in a linear transfer function that is sensitive to a wide-range of input amplitude (Figure 2C, noise).

Thus, in the presence of synaptic background activity, probabilistic “mixing” of single-spike and burst responses forms a multi-spike code, presumably controlled by the cortical input, that provides better encoding capabilities. It could enable TC cells to reliably detect and gradually respond to different degrees of input synchrony.

### Single-spike and burst responses at depolarized membrane potential rely on T-current

What could be the origin of the burst firing occurring in presence of synaptic bombardment in the depolarized state? Is it solely determined by synaptic inputs, or is the low-threshold T-current underlying the bursting mode also involved? We describe below that the activation of T-channels plays a major role in mixing single-spikes with bursts, and promotes responses with multiple spikes at depolarized membrane potentials where T-current was previously thought to be inactive (Deleuze et al., 2012).

*In vivo* cortical activity depolarizes TC neurons within a 10 mV range of potential, from approximately −70 mV up to −60 mV (Dossi et al., 1992). T-type calcium channels are classically thought to be fully inactivated in the -60 mV voltage range associated with the wake state (Coulter et al., 1989; Crunelli et al., 1989). Indeed, the role of T-channels has been restricted to rhythmic bursting during sleep or to occasional isolated bursts during sensory processing (Guido and Weyand, 1995; Ramcharan et al., 2000; Fanselow et al., 2001; Swadlow and Gusev, 2001; Martinez-Conde et al., 2002). Bursts were identified by a preceding period of silence, thought to be associated with hyperpolarization that deinactivate T-channels (Llinás and Steriade, 2006; Wang et al., 2007).

However, there is a high density of T-channels in TC cells (Bessaïh et al., 2008; Dreyfus et al., 2010) that far exceeds the number of channels required to generate a typical calcium spike following hyperpolarization. This excess of available channels results in a window T-current (Dreyfus et al., 2010), demonstrating the presence of a number of deinactivated T-channels in the −60 mV voltage range. We characterized the role of T-current in the transfer of sensory inputs using TTA-P2, a highly specific blocker of T-type channels (Dreyfus et al., 2010), and KO mice lacking the T-channel subunit expressed in thalamus. We show that available T-channels are involved in the boost of synaptic inputs and single-spike responses in the presence of background noise and explain the presence of burst responses to synaptic inputs seen in this depolarized state (Figures 2, 3).

We submitted somatosensory TC neurons to dynamic-clamp protocols similar to those described in Figure 1 under normal (control), blocked, and artificial T-current conditions. Neurons were maintained at strictly the same depolarized membrane potential (−58 mV), and dynamic-clamp sequences were replayed identically across all conditions (Figure 3A). In these highly controlled conditions that would be very challenging to obtain *in vivo*, we found that only the largest AMPA conductances are able to evoke firing when T-current is antagonized (Figure 3A, I_T_ block), as shown by the rightward shift of the corresponding input-output transfer function (Figure 3B, I_T_ block). This decrease in sensitivity when T-current is blocked is accompanied by a reduced burstiness. Not only the threshold to trigger single-spike responses becomes higher but the probability of generating burst responses drastically decreases (Figure 3C). In summary, this analysis shows that T-current promotes burstiness during wake-like depolarized potentials, enabling TC cells to integrate a range of sensory amplitudes in an efficient multi-spike code. Note that burst responses still occurs in the absence of T-current, and are very similar to the ones evoked in the presence of the T-current. Therefore, the involvement of T-current during bursts cannot be determined solely on the basis of the inter-spike intervals (see Deleuze et al., 2012 for details).

We demonstrated that the synaptic noisy input, presumably controlled by the cortex during focused attention, can tune the input-output transfer function of individual TC cells while promoting an efficient representation of sensory input amplitudes. In the brain, neurons are all different, and while the CT input must be topographically precise to enable directed activity modulations under attention, it is very unlikely that it is tuned in such a way as to accommodate the membrane properties of each TC cell. Therefore, there must be mechanisms responsible for the normalization of the input-output transfer functions. In the next section, we show that T-current has a determinant role in stabilizing the transfer function of TC cells across a range of membrane potentials, thus helping the cortex to exert its modulating influence across a diversity of neuronal properties.

### T-channel recruitment during synaptic noise stabilizes the transfer function across voltage changes

T-current not only boosts the tonic firing and burst occurrence at depolarized potentials but, due to its graded deinactivation with increasing hyperpolarization, also stabilizes the excitability of the neuronal population across a range of membrane voltages spanned by TC neurons during the waking state. We show here that the interaction of the T-current with background synaptic noise confers robustness to the response of TC neurons.

**Figure 4:**
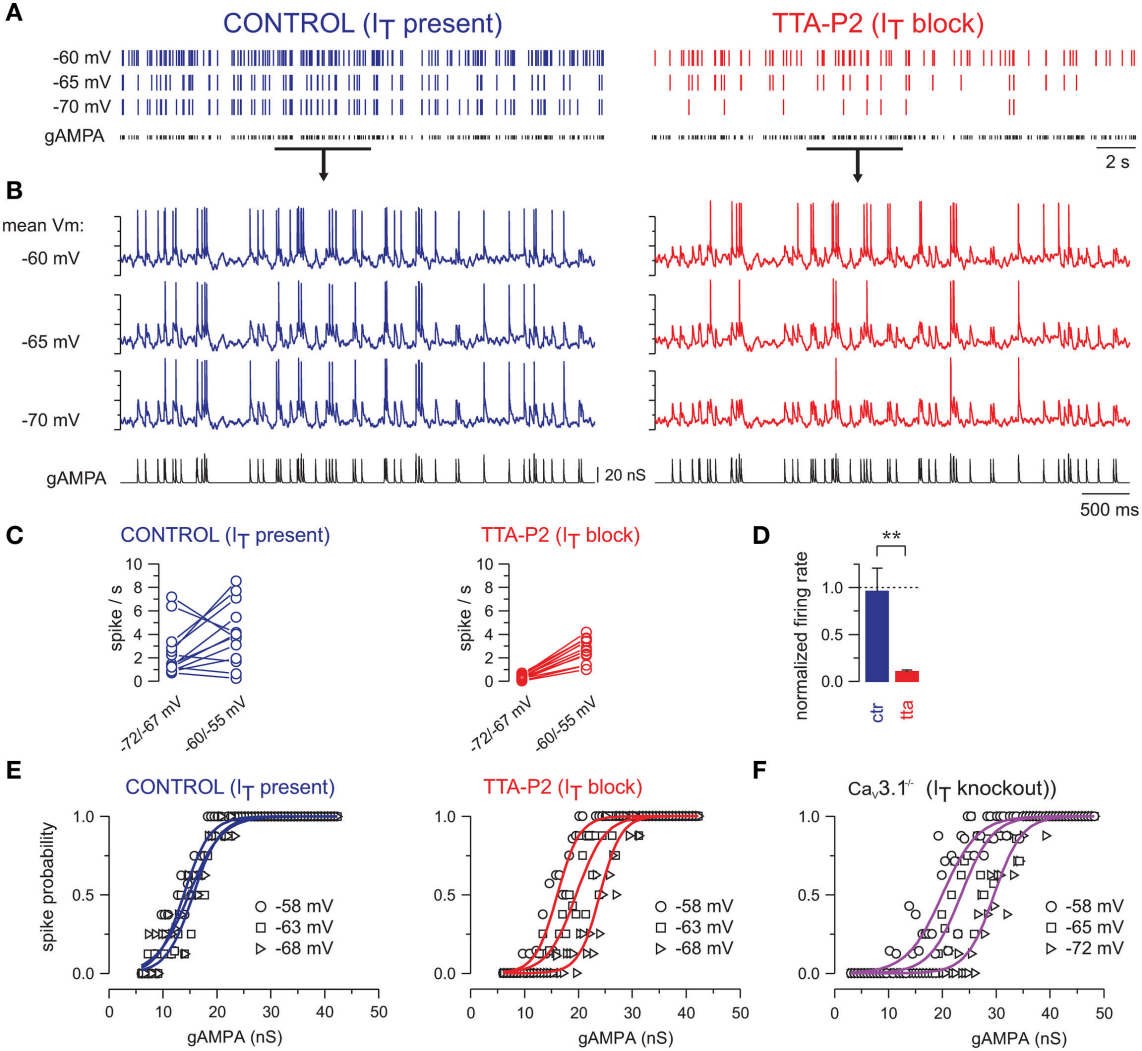
**T-current provides robustness to the response of TC neurons across a large range of membrane potentials**. **(A)** Spike raster plots of a TC neuron injected with gAMPA of fixed amplitude following a Poisson-distribution (mean frequency: 10 Hz), while the mean membrane potential was successively maintained at −60, −65, and −70 mV. **(B)** Intracellular activities recorded during the time windows indicated by the black line in **(A)**. In control condition the firing of the neuron remained almost invariant across the entire voltage range, but strongly decreased upon hyperpolarization when T-current was blocked. **(C)** Firing frequencies calculated in each neuron successively maintained at a membrane potential between −60/−55 mV and between −72/−67 mV while being submitted to the same gAMPA/noise sequences. Hyperpolarization induced either a decrease or an increase in firing frequency in control condition (CTR; *n* = 13) but a systematic decrease in the presence of TTA-P2 (TTA; *n* = 14). **(D)** In each neuron, the firing frequency at hyperpolarized potentials was normalized to the one at −60/−55 mV. Comparison of the mean values obtained with (CTR) and without T-current (TTA; ^**^p < 0.01; independent *t*-test) suggests that at the level of the TC neuronal population, T-channels rescued the voltage dependent decrease in firing induced by hyperpolarization. **(E)** Transfer functions were quasi-invariant in the presence of the T-current but drastically shifted toward larger gAMPA values upon hyperpolarization when the T-current was blocked. **(F)** Similar voltage dependence of the transfer functions was observed in TC neurons recorded in Cav3.1-/- knock-out mice devoid of T-current. **(E,F)** are from Deleuze et al. (2012).

The magnitude of a single retinogeniculate EPSP may vary little, but the effective retinogeniculate EPSPs depend on variable degrees of temporal summation, such that the effective input has a larger magnitude range (Turner et al., 1994; Usrey et al., 1998). Thus, Poisson-rate stimulation protocol allows varying the effective input EPSP magnitudes in a physiological way, as a result of summation (Turner et al., 1994). In Figure 4, TC neurons are successively maintained at different membrane potentials, ranging from −55 to −72 mV, while being submitted to the same temporal sequence of Poisson-distributed AMPA conductances and synaptic noise, in the presence of T-current or when T-current is blocked using TTA-P2.

When T-current is present, the evoked firing remains quite stable with only few spikes disappearing upon hyperpolarization (Figure 4A). In contrast, when T-current is blocked the firing responses of TC neurons are consistently and strongly decreased upon hyperpolarization (Figure 4B). Quantification of the neuronal firing with respect to two mean membrane potentials, i.e., −55/−60 mV and −67/−72 mV, shows a consistent decrease upon hyperpolarization when T-current is blocked (Figure 4C, right), whereas in presence of T-current, the neuronal firing increases in five neurons but decreases in the remaining eight neurons (Figure 4C, left). Overall, when considering the population of TC neurons, the mean firing rate remains almost stable throughout the 10 mV hyperpolarization in presence of T-current (Figure 4D; 96 ± 25% of the firing rate measured at depolarized potential; *n* = 13).

Therefore, our results suggest that T-current enables TC neurons to operate in a dynamic range of membrane potentials optimally. Another way to describe this property is that it maintains the neuronal sensitivity to sensory inputs stable across voltage changes, by stabilizing the transfer function of TC neurons when T-current is present (Figure 4E, control). In contrast, the transfer function of TC neurons is shifted toward a sensitivity to larger inputs upon hyperpolarization when T-current is blocked (Figure 4E, I_T_ block) or absent in mice that lack endogenous T-channels (Figure 4F).

In conclusion of this first part devoted to the integration properties of individual thalamocortical neurons, we show that synaptic noise tunes the integration of sensory input. Instead of a staircase-like input-output curve with limited coding capabilities for different input amplitudes, a linear response curve across the whole input range is generated, suggesting that, during synaptic noise, sensory signals can be relayed to the cortex with different efficiencies. Furthermore, the combination of synaptic noise with thalamic membrane properties generated by the T-current, gives a global responsiveness that is more stable at all membrane potentials, thus providing robustness to the thalamocortical transfer of sensory inputs. In the following we will consider how cortically-induced synaptic noise can control responsiveness at the higher level of integration of the thalamic cell assembly.

## A mechanism of top-down control of signal transmission emerges at the thalamic population level

### Convergence of thalamocortical neurons onto recipient cortical cells

Is it realistic to address the question of the efficiency of cortically-induced modulations of the thalamic sensory transfer solely from the interactions observed at the single-cell level, or does it emerge from higher order interactions within the network? There are important functional distinctions when considering either the isolated cell or the mesoscopic organization of a cell assembly. Our data obtained from experiments in individual cells, show that the background synaptic noise controls the cell responsiveness in a probabilistic manner, and the repetition of trials of similar inputs is necessary to average the response over time and build up the full description of the input-output transfer function. In the whole brain, the need for an immediate response makes trial averaging in individual cells impossible. Therefore, there must be mechanisms responsible for the rapid extraction of the probability function underlying neuronal responsiveness.

A large number of TC neurons, ranging from 15 to 125 in the cat (Alonso et al., 2001), form cell assemblies that converge onto individual recipient cortical neurons in primary visual cortex (Peters, 2002). We propose that this anatomical convergence of thalamocortical axons toward a recipient layer 4 cortical neuron is a critical circuit feature that allows a higher level of integration, where the targeted cortical neuron can decode the probabilistic signal integration distributed within its afferent thalamic circuit (Béhuret et al., 2013). In addition, the synaptic activity resulting from top-down cortical inputs can modulate this distributed integration process, thus providing a network-level mechanism for selective attention in the thalamus.

**Figure 5:**
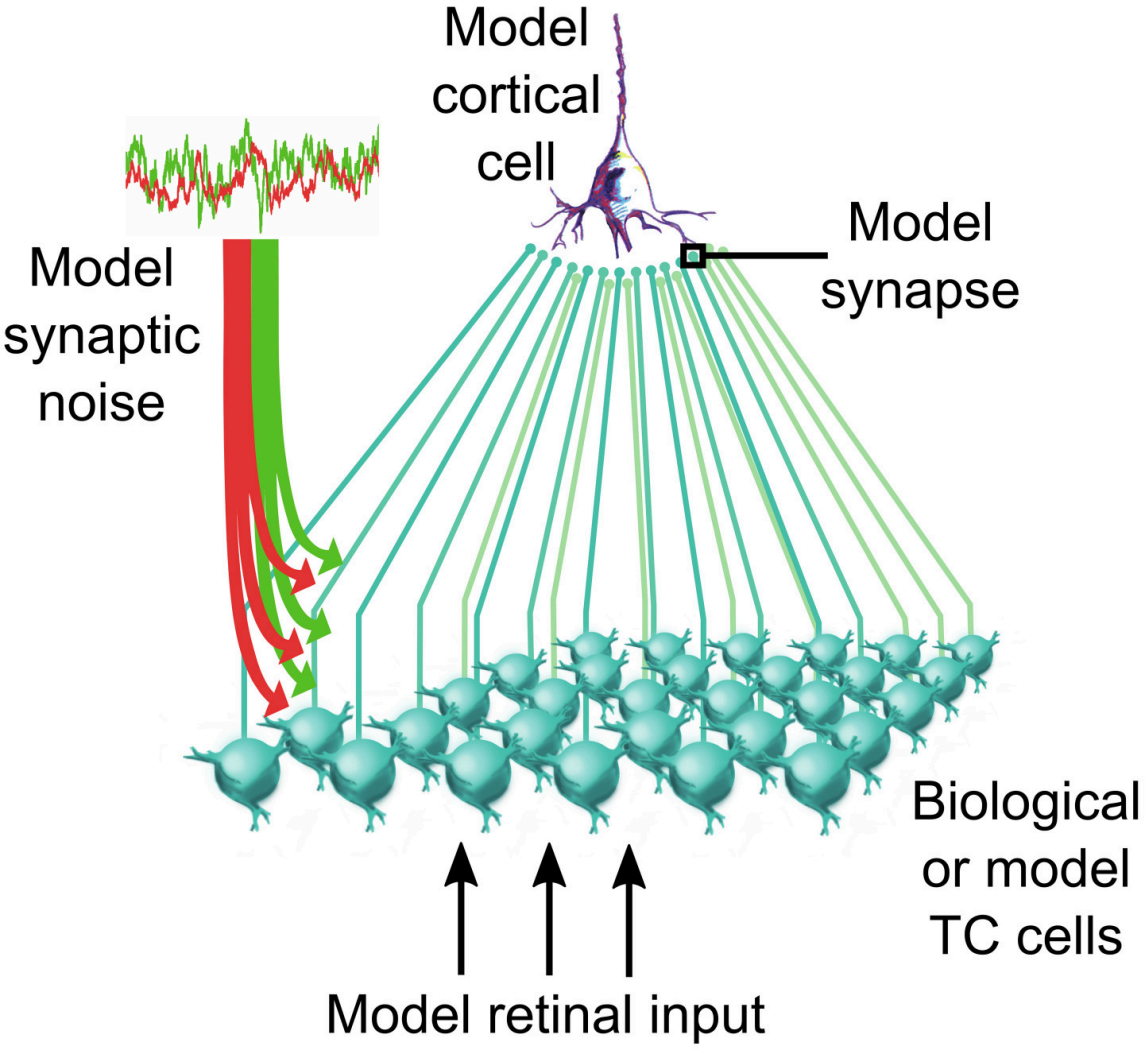
**Thalamocortical convergent circuit**. Biological or model TC cells network synaptically converge to a model recipient cortical neuron. The thalamic population receives a model retinal input in addition to a corticothalamic input mimicked through the injection of stochastically fluctuating mixed excitatory and inhibitory conductances. Details on the implementation of this circuit are available in Béhuret et al. (2013).

We tested this population-level control in networks of biological thalamic neurons forming a population of TC cells presynaptic to a recipient cortical cell (illustrated in Figure 5). We quantified the functional impact of the corticothalamic feedback on sensory information transfer in both computational model and iteratively constructed biological networks (Béhuret et al., 2013). In accordance with the dynamic-clamp paradigms used in our previous studies, biological TC cells were stimulated with retinal-like sensory inputs together with background synaptic noise mimicking the cortical input. But here, the method allowed not only to control critical parameters such as the mean and variance of the background conductances, but also the level of independence of the synaptic noise across thalamic cells.

### Background synaptic noise tunes the information transfer of sensory signals

As previously shown in individual TC cells, the input-output transfer function of retinal-like signals is measured as a spiking probability. In our model and biological networks of thalamic neurons, the transfer of sensory inputs to the receiver cortical cell is best captured with mutual information (Béhuret et al., 2013). We define the “transfer efficiency” as the transmitted information between the artificial retinal input and the cortical output spike-train. Application of the mutual information method to our network of one layer of neurons interposed between the input and the output is straightforward, and reflects faithfully the transfer properties of the circuit, when compared to other classical methods such as spike-transfer probability and spike-train cross-correlation analysis (see Figure S1 in Béhuret et al., 2013).

We varied the mean and variance of both excitatory and inhibitory components of synaptic noise, and found that the information transfer of sensory inputs is finely tuned by these two parameters at the level of the thalamic population. This effect results from an adjustment of the gain at the cellular level, where the spike response probability of each TC cell is shaped by the characteristics of the noise bombardment (Temereanca and Simons, 2004; Wolfart et al., 2005; Silver, 2010). The cumulation of gain adjustments at the population level further enabled the recipient cortical cell to integrate the converging thalamocortical lines and decode in a single trial the probabilistic function of presynaptic TC cells lumped together.

**Figure 6:**
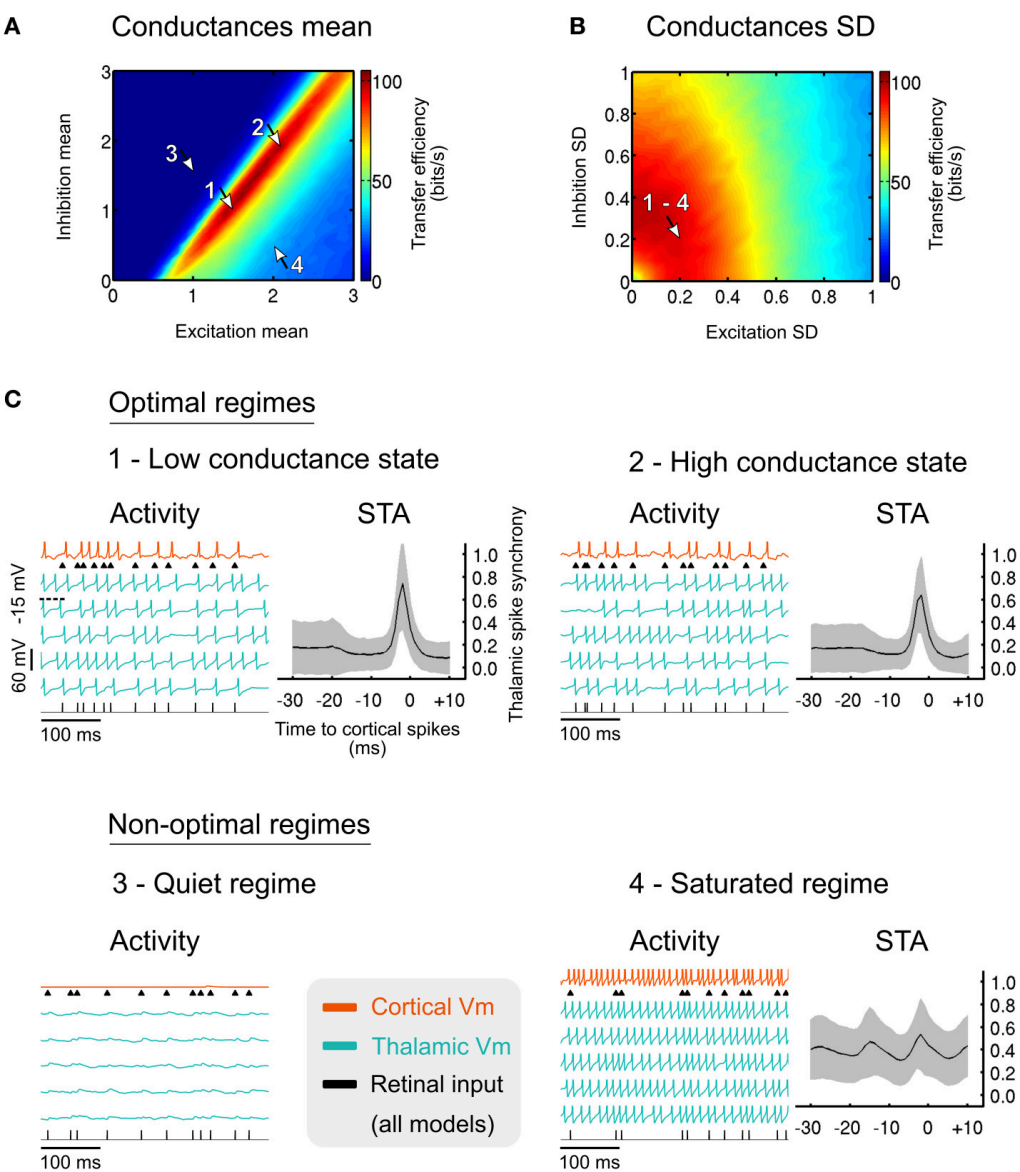
**(A)** Effect of the cortical excitatory and inhibitory mean input conductances on the sensory transfer efficiency. Mean conductances were normalized relative to the resting conductance of the thalamic cells. Arrows indicate specific operating regimes illustrated in **(C)**. The normalized conductance standard deviations were normalized relative to their respective means, and set to 0.2 for both excitation and inhibition. **(B)** Similar to **(A)** for the standard deviation of the excitatory and inhibitory conductances. The normalized conductance means were set to 1.5 for the excitation and 1.0 for the inhibition. **(C)** Membrane voltage traces for the four specific regimes denoted by the arrows in **(A,B)**. The thalamic spike synchrony was measured with cortical spike-triggered average. The number of thalamic spikes evoked in the corresponding regimes was averaged using a bin size of 1 ms and was then normalized to the total number of TC cells. Grayed areas represent the standard deviation of the counts across all cortical spikes (*n* > 10^3^ in every bin). Modified from Béhuret et al. (2013).

A systematic exploration of the parametric space allowed us to determine optimal synaptic noise parameters, corresponding to the maximization of the sensory transfer efficiency by means of mutual information. Optimal noises generated by cortical synaptic inputs are revealed by the elongated hot spot of efficient transfers in Figure 6A, and are characterized by quasi-balanced levels of excitation and inhibition over a wide range of conductance states. Small conductance fluctuation amplitudes, generating small voltage fluctuations ranging from 1.0 to 1.4 mV in thalamic neurons (standard deviation after removal of spikes), is optimal for high transfer efficiency (Figure 6B). Therefore, small noisy fluctuations in membrane potentials observed *in vivo*, rather than being insignificant, could in fact reflect such mechanism of population gain control.

The anatomical convergence of thalamocortical synapses to a receiver cortical cell is adapted to detect thalamic synchrony. This can be seen in Figure 6C where typical activity regimes and their corresponding cortical spike-triggered averages (STA) are shown. In the optimal regimes (arrow 1 and 2 in Figures 6A,B), the STA show an increase of the thalamic input synchrony a few milliseconds before cortical spikes. Low and high conductance states, which represent different levels of conductance input strength, lead to similar activity regimes and are both as effective for the relay of sensory information (see Discussion). In the silent regime (arrow 3), no spikes are evoked due to the concomitant action of strong inhibition and weak excitation. In the saturated regime (arrow 4), thalamic and cortical neurons are firing in a tonic mode due to a saturating level of excitation, resulting in a non-specific cortical STA. The above results suggest that optimally tuned background synaptic noise, as reflected by the hot spots in Figures 6A,B, facilitates the synchronization of sensory-evoked thalamic spikes, and hence their detection by the receiver cortical cell. In other regimes, the synchronization of thalamic spikes is prevented, which further decouples the cortical spikes from the retinal input.

Given these results, the relay of sensory features encoded by presynaptic thalamic populations could depend on the precise timing of thalamic spikes. During successful transfer of retinal spikes, the synchronization of thalamic spikes falls within a ~10 ms time window (Figure 6C, optimal regimes STA), which is consistent with the spiking opportunity window for thalamic spikes (Pouille and Scanziani, 2001), the thalamic synchronization tuning resulting from adaptation (Wang et al., 2010), and retinogeniculate paired-spike enhancements (Usrey et al., 1998; Kara and Reid, 2003). This view is also confirmed by a recent study that demonstrates the importance of thalamic synchrony for cortical feature selectivity, with the most efficient transmission at a level of thalamic synchrony in the range of 10–20 ms (Kelly et al., 2014).

Therefore, our results indicate that the cortical feedback tunes the sensory information transfer by switching thalamocortical activity regimes, which has an impact on the firing rate and synchrony of thalamic spikes. This modulatory effect observed at the population level may account for the modulation of sensory transfer in the thalamus during attention. However, the mechanisms implementing selective attention at the circuit level may not be as straightforward as a firing rate modulation, as it is suggested by studies showing changes in activity correlations during focused attention (see Introduction). In the following simulations and experiments we investigated the functional impact of synaptic noise (de-)correlation across TC cells, a critical feature in the control of information transfer.

### Synaptic noise decorrelation boosts sensory information transfer

**Figure 7:**
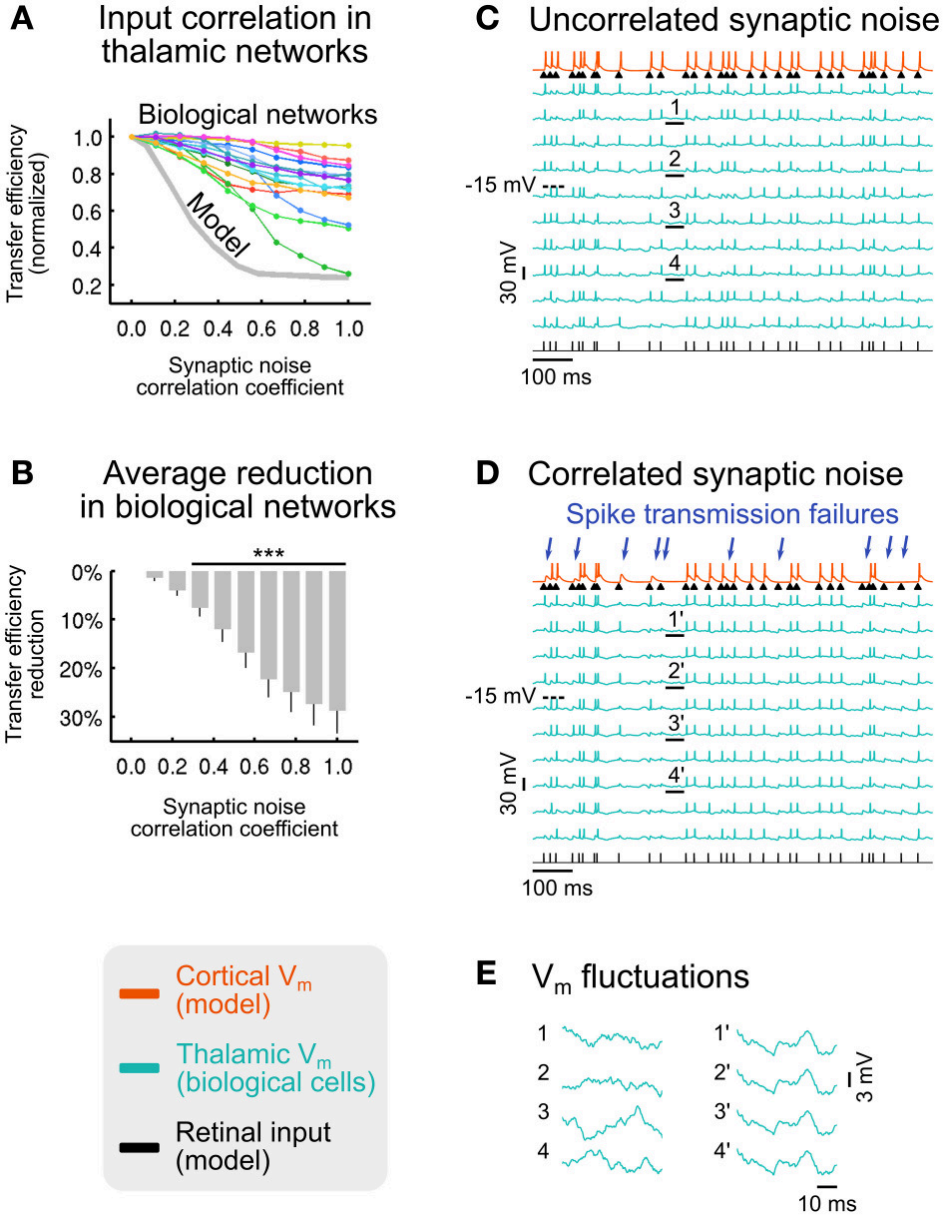
**Effect of synaptic noise correlation across TC cells on sensory information transfer**. **(A)** Normalized transfer efficiency in model (gray curve) and biological networks (colored curves) for increasing levels of synaptic noise correlation across thalamic neurons. **(B)** Average transfer efficiency reduction (± SEM) across all biological networks shown in **(A)** (^***^p < 3.10^−4^; *t*-test; *n* = 15). **(C)** Illustration of voltage traces for a biological network receiving uncorrelated synaptic bombardment. **(D)** Same biological network as in **(C)** receiving correlated synaptic bombardment. Retinal spikes that were detected by the recipient cortical neuron in **(C)** but not detected in **(D)** are indicated by blue arrows. **(E)** Zoomed sections of membrane potential fluctuations underlined in **(C)** (sections 1–4; uncorrelated synaptic bombardment) and **(D)** (sections 1'–4'; correlated synaptic bombardment). Modified from Béhuret et al. (2013).

To explore the effects of synaptic noise (de-)correlation, we imposed a range of correlation levels in the synaptic bombardment across TC cells, ranging from complete desynchronization, as in the previous parts of this study, to full synchronization, in the model (Figure 7A, gray curve) and in networks of biological thalamic neurons (Figure 7A, colored curves). We found that the sensory transfer efficiency decreases with increasing levels of correlation in the synaptic noise. Desynchronized (uncorrelated) top-down input was thus highly efficient in promoting retinal signals transfer to the recipient cortical neuron, while correlated input has the opposite effect of strongly reducing the relay by up to 76%. As seen throughout our analysis, background noise (de-)correlation is not an all-or-none “permissive” mechanism, suggesting that the brain may be able to gradually adjust the information transfer of selected sensory signals. Furthermore, the retinocortical transfer is not entirely switched off even with a fully correlated corticothalamic synaptic bombardment.

The average transfer efficiency reduction in the tested biological networks becomes highly significant for correlation coefficients larger than 0.33 (Figure 7B), suggesting that low correlation levels have a strong impact during sensory processing. We also explored in model circuits how the correlations imposed in the top-down input affected the correlations between thalamic spikes. In model circuits, a synaptic noise correlation of 0.28 had the minor effect of increasing the pairwise correlations of thalamic spiking activity from ~3 to ~11%, while imposing a major reduction of the transfer efficiency by more than 50%. This analysis shows that differences in thalamic pairwise spike correlations, so small that they may not be detected using dual recordings *in vivo*, can nonetheless strongly impact thalamocortical processing. Our results are consistent with data showing that neurons with similar orientation tuning in the primary visual cortex of awake macaques virtually share no correlation (Ecker et al., 2010), and a study stressing the high impact of the low correlations in neural populations (Schneidman et al., 2006).

The deleterious effect of synaptic noise correlation can also be seen on activity traces. We found that, when compared to the uncorrelated condition (Figure 7C), ~30% of retinal spikes were not detected by the recipient cortical neuron in the correlated synaptic bombardment condition (Figure 7D), although the thalamic firing rate was nearly identical in both conditions (uncorrelated: 20.3 Hz; correlated: 21.0 Hz). This is reflected by a significant reduction of the retino-cortical spike transfer probability, from 0.97 to 0.75 (average across non-overlapping activity windows; *p* < 10^−7^; paired-sample *t*-test; *n* = 10).

These analyses in model and biological networks point out the determinant role of synaptic noise decorrelation in the transfer of sensory signals. However, we have seen in the previous section that optimal background noise facilitates the synchrony of thalamic spikes, but how can this aspect be reconciled with the positive effects of noise decorrelation? When synaptic bombardment is highly correlated across the thalamic population, sensory-evoked thalamic spikes are either amplified or attenuated simultaneously in every TC cell (Figure 7D) as a result of membrane voltage fluctuations being nearly identical on the basis of similar intrinsic membrane properties (Figure 7E, right traces). Although this configuration leads to highly synchronous thalamic spikes, this uniformization of the thalamic population response is detrimental for the transfer of sensory information. We explain this counter-intuitive effect in the following way. Strong depolarizations randomly triggered by the correlated top-down noise sometimes lead to non-sensory spikes in every TC cell simultaneously, and further transmit retina-unrelated spikes to the receiver cortical cell. Similarly, randomly induced top-down hyperpolarizations in register with retinal spikes sometimes prevent TC cells from responding. Therefore, correlated background inputs, which result in an error-prone spiking behavior across the thalamic population, have deleterious effects on the transfer of sensory signals, and substantially decouple the causal link between the retinal input and the cortical output. Conversely, weakly correlated or uncorrelated inputs promotes a diversity of subthreshold thalamic responses (Figure 7E, left traces), with each thalamic spike being independently amplified or attenuated by synaptic noise (Figure 7C). This stochastic resonance property of synaptic noise (Rudolph and Destexhe, 2001; McDonnell and Abbott, 2009) allows each presynaptic TC cell to independently detect sensory signals for which the recipient cortical cell is selective, and results collectively in a stochastic facilitation process (McDonnell and Ward, 2011) across the thalamic population.

In summary, the exploration of information transfer properties in model and biological networks reveals that decorrelation of the synaptic bombardment facilitates the transfer of sensory signals to the cortex, an effect which only emerges from the collective action of TC cells. In light of these data, the decorrelation of the CT input appears to be a potent mechanism by which the neocortex could exert its action on sensory inputs. This view is also supported by a recent modeling study suggesting that the corticogeniculate feedback enables efficient representations of input information by decorrelating LGN responses (Zabbah et al., 2014). Combined with a dynamic modulation of the mean and variance of the cortical feedback input, these mechanisms could be used by the brain to actively filter the sensory information that is conveyed by retinal ganglion cells, reflecting both attentional processes and active stimulus filtering under the supervision of cortical areas.

We finally considered an extreme mode of correlation, largely present in the brain in the form of widespread synchronized oscillations of various but specific frequencies, that are known to impair signal transfer during sleep (Le Masson et al., 2002; Dang-Vu et al., 2010) and absence epilepsy (Hughes, 2009), promote loss of consciousness (Ching et al., 2010), and show reduced magnitude during focal attention (Bollimunta et al., 2011). We investigated to which extent such oscillation-induced correlations imposed in the convergent structure of the thalamic network would affect signal transmission.

### Synchronized oscillations in the thalamus block sensory information

Thalamocortical oscillations are stereotyped in frequency and amplitude, lack the broadband variability of the cortical noise and are widely present in the thalamocortical system during wakefulness and sleep. During relaxed wakefulness, the electroencephalogram (EEG) exhibits robust rhythms in the alpha band (8–13 Hz), which decelerate to theta (2–7 Hz) frequencies during early sleep (Hughes et al., 2004; Hughes and Crunelli, 2007), followed by the 10–14 Hz spindles waves and the slow (< 1 Hz) rhythms during non-REM sleep (Steriade et al., 1993; for a review see Crunelli and Hughes, 2010). We proposed earlier that spindles, which are perhaps among the best-understood synchronized oscillations generated endogenously in the thalamocortical system during slow wave sleep (von Krosigk et al., 1993; for a review see McCormick and Bal, 1997), have the property of imposing a temporal decorrelation of retinal input and thalamic relay output, resulting in the functional disconnection of the cortex from the sensory drive (Le Masson et al., 2002). Conversely, during wakefulness, the waning of synchronized oscillations, and in particular the decrease of power in the alpha (Bollimunta et al., 2011) and mu (Jones et al., 2010) bands seem to be associated with attention. Altogether these data suggest that thalamocortical oscillations are involved in sensory filtering during attention.

**Figure 8:**
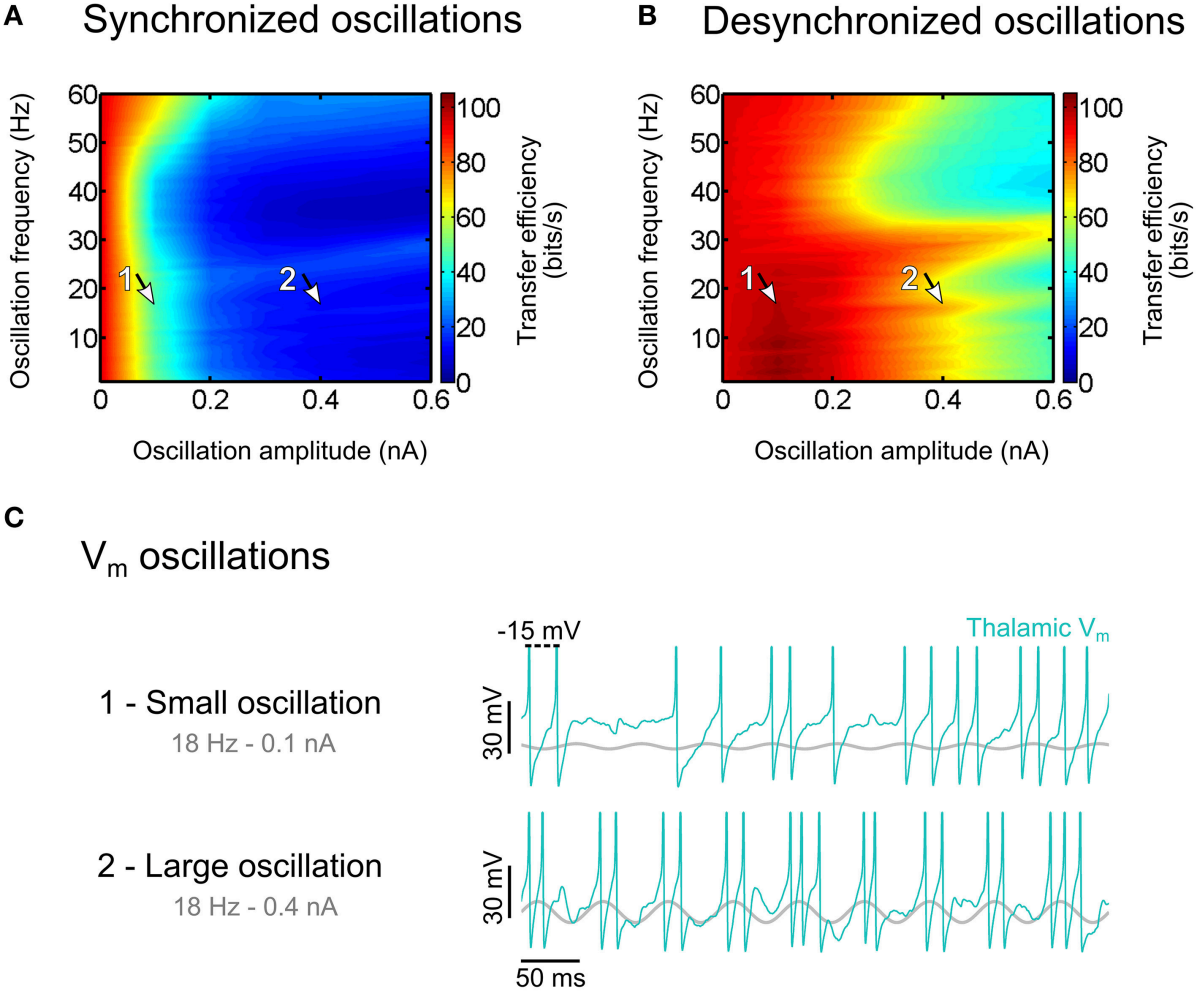
**Effect of thalamic oscillations on sensory information transfer**. **(A)** Transfer efficiency for synchronized oscillations. **(B)** Same as **(A)** for desynchronized oscillations. **(C)** Thalamic membrane potential traces for oscillation amplitudes of 0.1 nA [arrow 1 in **(A)**, degraded transfer] and 0.4 nA [arrow 2 in **(B)**, permissive transfer]. Modified from Béhuret et al. (2013).

We induced oscillations in model thalamic populations by injecting sine-wave currents of varying amplitude and frequency in relay cells, in addition to retinal-like sensory inputs and uncorrelated synaptic bombardment. We found that imposing synchronized oscillations across the thalamic population results in a large decrease of the sensory transfer efficiency (Figure 8A). In contrast, desynchronized oscillations, where the phase is homogeneously distributed across the thalamic population, have only a minimal effect on the transfer efficiency (Figure 8B).

Small oscillation amplitudes that did not produce any visible rhythmic activity in the membrane potentials of relay cells (Figure 8C, top trace for 0.1 nA) largely degraded the transfer efficiency in the synchronized condition (arrow 1 in Figure 8A). By contrast, larger oscillation amplitudes that substantially entrained thalamic membrane potentials (Figure 8C, bottom trace for 0.4 nA), had only a minor impact on the sensory transfer property in the desynchronized condition (arrow 2 in Figure 8B). This comparison between small synchronized and large desynchronized oscillations emphasizes the detrimental effect of modulatory input correlation on sensory transfer, even for oscillation amplitudes so small that they could barely be detected in membrane potential traces.

Our results provide new insight into the role of coherent oscillations in the thalamocortical system. Oscillations may be used by the brain as an effective way to regulate the information transfer of sensory signals. This can be seen with sleep spindles, which are associated to sleep robustness. Spindles are spatially correlated in the thalamocortical system (Contreras et al., 1996), and EEG data show that people having more spindles during sleep are more likely to stay asleep in noisy situations (Dang-Vu et al., 2010), suggesting that spindle oscillations block auditory signals. Signal decoupling by means of synchronized oscillations is most likely to reach its maximal impact in situations of anesthesia or epilepsy. A theoretical study based on human EEG recordings proposed that the thalamocortical coherence induced by Propofol, a short-acting hypnotic agent, is a generative mechanism for the loss of conscious sensory experience (Ching et al., 2010). It is possible that synchronized oscillations in the alpha band are part of an active attentional suppression mechanism aimed at ignoring irrelevant or distracting information (Foxe and Snyder, 2011). Interestingly, it is proposed that this suppression mechanism plays a key role in filtering inputs to primary sensory neocortex, and can be improved in cognitive therapies based on mindfulness meditation (Kerr et al., 2013). Standardized mindfulness alleviates chronic pain and depression relapses, and this could result from optimized attentional modulation of 7–14 Hz alpha rhythms.

The overall evidence suggests that synchronized oscillations may be used by the brain to set the thalamic gateway into a default, non-permissive oscillatory regime, limiting the transfer of sensory signals during sleep, and perhaps during wakefulness, in thalamic regions not attended by the cortical feedback. In the next section, we propose a phenomenological model of selective attention explaining how the cortical feedback may become decorrelated, modulate the spatial coherence of thalamic oscillations, and boost the signal transfer of attended sensory features.

### Selective attention: a feedback decorrelation model

**Figure 9:**
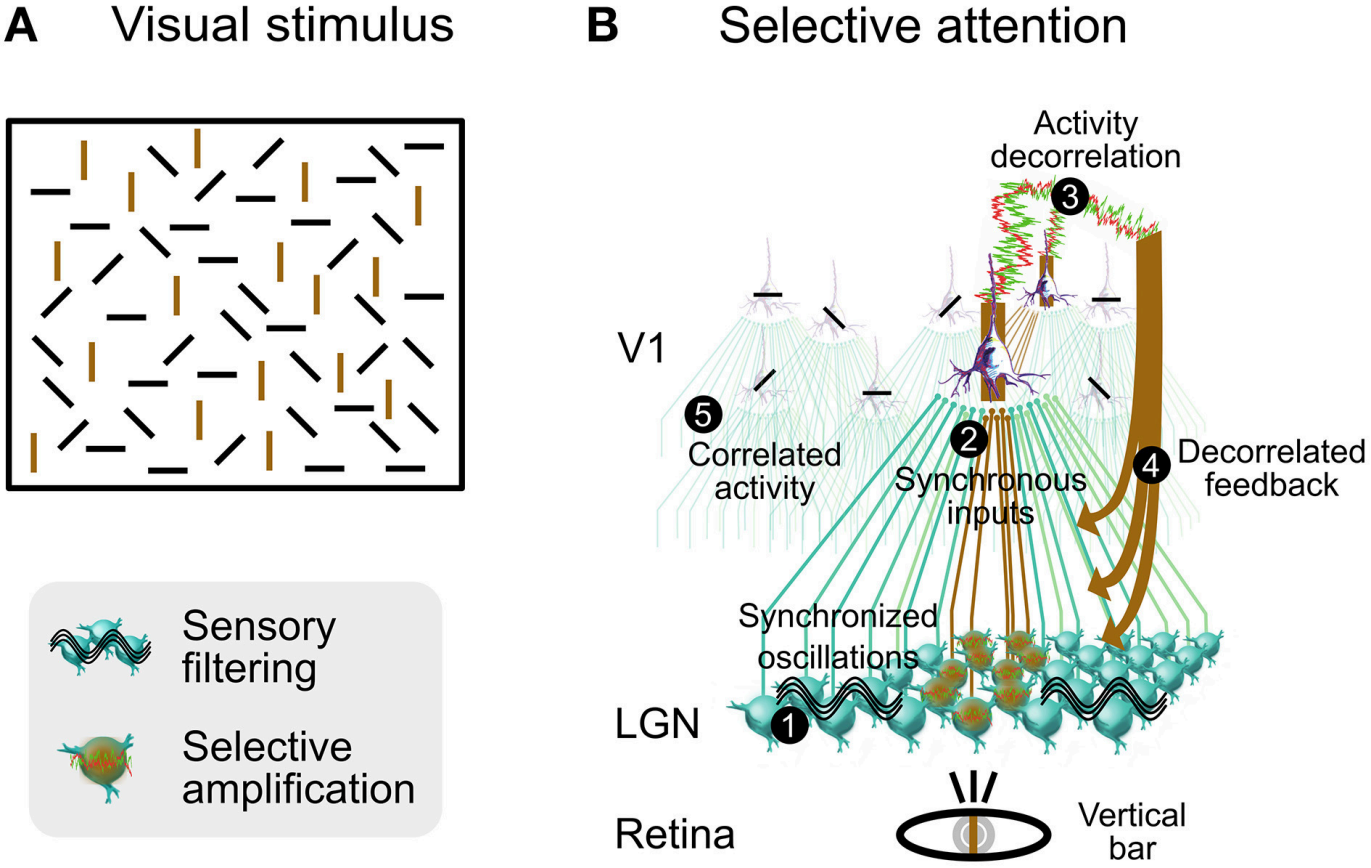
**Speculative role of synaptic bombardment decorrelation and thalamic oscillations in selective attention**. **(A)** Visual stimulation composed of bars of various orientation. Focusing attention on a single bar (for instance vertical) will slowly segregate all other bars of same orientation from the context made of other bars of dissimilar orientation. Vertical bars are colored in brown for illustration purposes only. **(B)** Presumed functional steps involved when focusing attention on a vertical bar (see text for details). Bars shown on each neuron illustrate the orientation preference. Columnar organization of V1 circuits is not illustrated although each cortical neuron shown in this schema belongs to a different orientation column. Modified from Béhuret et al. (2013).

We illustrate how selective attention may involve synaptic noise decorrelation in a functional scenario consisting of a visual stimulus composed of bars of four different orientations (Figure 9A). When our attention is focused on a single bar (for instance vertical), then all other bars of same orientation are segregated from the context made of other bars of dissimilar orientation and become selectively perceived. This effect is rather slow and demands an attentional effort, unlike the classical pop-out that is automatic and fast (Hochstein and Ahissar, 2002). This slower perception process could depend on a feedback cascade from higher-level cortical areas, and on horizontal synaptic connections in V1. Note that the color of the bar is not an issue here, the effect is the same when bars are all of the same color.

We propose that a decorrelated cortical feedback modulates the spatial coherence of thalamic oscillations in regions that are retinotopically aligned with bars similar to the one being focused, thus boosting their sensory transfer to the cortex. Based upon this decorrelation mechanism, we describe a phenomenological model in several steps (Figure 9B) for this visual effect, produced by contrasting levels of activity correlation between V1 columns selective to the orientation of the attended bar and other columns:

**- Initial State of the Thalamic Gateway**: Oscillations in the thalamus are generated by various known mechanisms such as intrinsic properties, gap junctions and synaptic interactions with NRT (McCormick and Bal, 1997; Hughes et al., 2004; Lorincz et al., 2009). They are synchronized in the alpha-beta range between V1 and dLGN during attention (Bekisz and Wróbel, 2003), an effect which may rely on corticothalamic projections (Contreras et al., 1996). In this state the thalamic gateway is marginally permissive to sensory signals, and highly synchronized sensory input and the activation of T-type channels may be necessary to reliably relay sensory information to the neocortex.
**- Synchronous Sensory Inputs to Cortical Area V1:** The contour of the focused bar is detected by retinal ganglion cells, which further discharge synchronously. In the retinotopic stream activated by the attended vertical bar, thalamocortical relay cells are activated by the synchronous retinal inputs, and activate cortical cells that are selective to the orientation of the focused bar.
**- Decorrelation of Activity in Cortical Area V1 in Same-Orientation Columns:** Sensory input to V1 area leads to activity decorrelation (Renart et al., 2010; Middleton et al., 2012), switching visual cortex from synchronous to asynchronous states (Tan et al., 2014), and feedback from high-level visual areas may contribute to or reinforce this decorrelation (Hochstein and Ahissar, 2002; Greenberg et al., 2012). We predict that an LFP electrode precisely located within the target region of the focused attention in V1 would reveal decreased levels of correlation in the alpha-beta or gamma frequency ranges or both. Decorrelated activity propagates to other columns via connections between columns of same orientation (Bosking et al., 1997; Angelucci et al., 2002; Lund et al., 2003). At this stage, the emerging activity in V1 is characterized by decorrelated activity in columns that are selective to an orientation matching that of the attended bar, and by correlated activity in columns selective to other orientations. Correlated activity could be further amplified by the lack of thalamocortical input resulting from the underlying filtering of sensory information (this notion of sensory filtering is explained in step 5).
**- Selective Amplification of Sensory Signals by Foci of Corticothalamic Decorrelation:** Regions of decorrelated activity in V1 send a decorrelated corticothalamic synaptic bombardment to their target neurons in the dLGN and in the NRT, disrupting the synchronization of thalamocortical oscillations, and boosting the sensory transfer at these specific locations. We infer from the retinotopic organization of the top-down projections that the target neurons in the dLGN are specifically tuned to detect features matching bars with an orientation similar to the one being focused.
**- Filtering of Irrelevant Information by Synchronized Activity:** In regions aside from decorrelated corticothalamic foci, the descending cortical input remains correlated. At these locations, thalamic oscillations and correlated feedback impose a reduction in the transfer efficiency of sensory signals that are unrelated to the focused bar and to other bars of similar orientation. High correlations in V1 are essentially sustained by horizontal activity, intracortical recurrent activity, and thalamocortical oscillations. For instance, increased synchronization in the gamma frequency range may stem from elevated oscillatory retinal frequencies (Troy and Robson, 2009), intracortical mechanisms including high-frequency bursting (Gray and McCormick, 1996) and interneurons activity (Traub et al., 1998). Likewise, synchronization in the alpha-beta frequency range may be driven by intrinsic thalamic oscillations described in step 1.

The originality of our proposal is that, for a given attentional task, correlated and decorrelated activities coexist, which is consistent with studies reporting either increased or decreased correlations in the alert animals and specific correlation patterns in humans performing attentive tasks (see Introduction). Thalamocortical regions being the focus of selective attention would present low correlations, while other regions would present higher correlations, possibly sustained by a default, non-permissive oscillatory regime of the thalamic gateway. An experimental consequence would be that detection of increased or decreased correlations in neuronal activity should entirely depend upon the location of an LFP electrode, which picks up signals from restricted cell populations, and primarily reflects synaptic activity local coherence. We therefore predict the existence of dynamic functional maps of correlation and decorrelation in V1 and in the thalamus that reflect the deployment of attention in the early visual system. These maps differ from classical cortical maps, such as those of orientation and ocular dominance in V1, in that they should vary according to the stimuli and the attentional context, and their distribution should change from moment to moment.

In summary, we envision a mechanism of selective sensory attention by which top-down cortical inputs could create an ever and rapidly changing landscape of islands of highly efficient sensory spike transfer in a network otherwise functionally decoupled from its inputs.

## Discussion

### Synaptic noise determines the transfer of sensory signals in the thalamus

In this paper we summarize our recent findings supporting the hypothesis that corticothalamic synaptic activity is adapted to modulate the sensory transfer of thalamocortical neurons at different levels. At the ionic channel and neuronal levels we show that the noisy high-conductance state, mixing inhibition and excitation, that neurons experience *in vivo* (Steriade, 2001; Destexhe et al., 2003) interacts with neuronal built-in integrative properties and influences the transfer function of individual TC neurons (Wolfart et al., 2005; Deleuze et al., 2012). At the network level, the axonal projections of many thalamocortical neurons converge onto individual cortical neurons, enabling receiver cortical neurons to collect many thalamocortical inputs, each of them firing one or several spikes in response to sensory input. We show *in vitro* and *in computo* (Béhuret et al., 2013) that this circuit feature endows the receiver cortical cell with the capability to extract the response probability function of the thalamic cell assembly. We found that this integrative property is under the tuning control of synaptic noise. Importantly, at the thalamic population level, the stochastic facilitation of sensory transfer emerges via a cortical mechanism of decorrelation of subthreshold synaptic noise and oscillations.

Activity desynchronization may reflect an active computing principle for the selection of sensory information during attention, which is suggested by studies showing reduced interneuronal correlations in visual area V4 of monkeys performing an attentive task (Reynolds et al., 2000; Cohen and Maunsell, 2009; Mitchell et al., 2009) (for a review, see Reynolds and Chelazzi, 2004). For instance, multiple units recording in this area revealed that fluctuations in firing rate, which are correlated across relatively large populations of neurons, are reduced by spatially selective attention (Cohen and Maunsell, 2009; Mitchell et al., 2009). These attention-dependent reductions in correlated firing could produce a far greater improvement in signal-to-noise ratio than increases in firing rate associated with attention would do (Mitchell et al., 2009). This view is also supported by a number of experimental and theoretical studies pointing to the importance of activity desynchronization for an improved sensory processing (Cohen and Maunsell, 2009; Mitchell et al., 2009; Ching et al., 2010; Dang-Vu et al., 2010; Jones et al., 2010; Bollimunta et al., 2011; Béhuret et al., 2013).

Several non-exclusive mechanisms may also contribute to selective attention via permissive or suppressive action on sensory transfer in the thalamus (Casagrande et al., 2005). The neuromodulation of membrane properties of relay cells is part of them and it has the potential of strongly changing the input resistance of the cells via tonic synaptic activities. The activity of afferents coming from brainstem triggers neurotransmitters release in the thalamus (acetylcholine, noradrenaline, etc.), which contributes to synaptic noise and modulation of membrane properties. These synaptic influences depolarize thalamic relay neurons in a voltage range at which rhythmic oscillations are not prevalent, and promotes a single spike activity as well as enhanced sensory responses (McCormick, 1992). We had previously shown in hybrid circuits that application of noradrenaline increased both efficiency and reliability of retinal spike transfer to cortex (Le Masson et al., 2002). The neuromodulation course of action however does not seem to match the spatial and temporal precision needed for visual attention (Casagrande et al., 2005). In contrast, the cortical control of thalamic signal transfer by a tunable mixed excitatory and inhibitory synaptic background activity, as proposed in this paper, presents several advantages over the modulation by neuromodulators: It is dynamic, fast, and topographically precise.

### Impact of synaptic noise on membrane properties

By injecting stochastic conductances into thalamocortical neurons, we show that the transfer function of TC neurons is strongly influenced by conductance noise. We propose that the duality of spiking modes in thalamic relay neurons, with bursting during sleep and tonic single-spikes firing during wake, no longer holds if synaptic background activity is taken into account. During sleep, thalamocortical cell bursting is part of a large-scale synchronized activity, and this “proper” burst mode transmits state-dependent information to the cortex, different from the information transferred by single spikes (McCormick and Bal, 1997; Sherman, 2001; Steriade, 2001). However, during activated states when TC neurons receive background synaptic inputs, single-spike and burst responses may both contribute to an efficient encoding of visual information (Reinagel et al., 1999). Thus, the co-occurence of single-spikes and bursts in response to excitatory stimuli, as well as the more graded aspect of bursts in a multi-spike code, suggest that with background synaptic activity, there is indeed no clear distinction between single spikes and bursts.

The combined effect at the network level of synaptic noise acting on individual TC neurons promotes an efficient information transfer of sensory inputs to the cortex. Peak transfer efficiencies are obtained for nearly balanced regimes of excitation and inhibition, with a total synaptic noise conductance ranging from a low to a high conductance state. The conductance state commonly refers to the strength of the synaptic input. Although the functional distinction between the low and high conductance states is not apparent from the presented results, it is indeed an important one. In high conductance states, synaptic noise is characterized by larger conductances and has a stronger modulating influence on voltage fluctuations. In our protocols there was no extra-cortical competitive input to the thalamic layer, therefore both low and high conductances states led to similar voltage fluctuations in TC neurons. However, in presence of competitive inputs, synaptic noise in a high conductance state would drive the voltage dynamics of TC cells more effectively. Thus, in the intact brain where TC neurons receive additional inputs from regions of the brainstem and basal forebrain (Sherman and Guillery, 1996), it is possible that the conductance state due to synaptic noise forms yet another channel by which the cortex exerts its influence on sensory transfer, with a maximal modulating strength reached during the high conductance state.

We show that the T-current strengthens the input-output relationship of sensory-like input of every TC neuron studied, in full agreement with a recent study showing that T-current underlies the homeostasis of the input-output relation (Hong et al., 2014). Furthermore, we found a cell-to-cell variability in the stability of the neuronal response over membrane voltages. This heterogeneity may originate from the different T-current densities that are expressed in each neuron; this possibility remains to be tested experimentally. Large differences in neuronal properties can interfere with information processing in thalamic networks, yet some degree of variability is beneficial for sensory signal transmission to the cortex, an effect which relies on the decorrelation of sensory responses across neurons receiving shared inputs (Béhuret et al., 2013). This view is also supported by a study in the olfactory bulb glomerulus showing that variability in intrinsic properties of mitral cells increases the information content of the cell population (Padmanabhan and Urban, 2010; Wilson, 2010).

Thus, cell-to-cell differences in T-current density among the thalamocortical population appear to be valuable to information processing at a population level, and may contribute to the optimization of sensory transfer to the cortex. However, alteration of cellular biophysical properties is a slow adaptive process (Nelson and Turrigiano, 2008) which is presumably not suited to achieve fast and reversible dynamical regulation of sensory transfer. In contrast, input synaptic variability is a dynamical process governed by the presynaptic activity of thousands of neurons and is capable of adapting rapidly, perhaps instantaneously, to the needs of sensory transfer. Because in thalamic neurons, background synaptic input originates mainly from the cortex, these results support a determinant role of cortical layer 6 for the control of sensory information transfer across the thalamic gateway.

### The elusive activity of layer 6 corticothalamic neurons

In regard to the high degree of receptive field specificity of layer 6 neurons (Swadlow and Weyand, 1987) and of their massive projections to the thalamus (and to other cortical layers), it has been a long-standing enigma that these neurons were often found largely unresponsive or firing at low rate in the anesthetized (Kelly et al., 2001) and especially in the awake animal (Swadlow and Weyand, 1987; Swadlow, 1989; O'Connor et al., 2010).

A few studies suggest that the corticothalamic feedback is engaged in the behaving animals. Inputs to the primary sensory cortex from another functionally related cortical area are critical to alter the excitability of corticothalamic neurons in lightly sedated rats (Lee et al., 2008), where the enhancement of firing in deep layers of motor cortex facilitates whisker-evoked responses in both topographically aligned S1 corticothalamic neurons and thalamic VPm neurons. In mouse V1, layer 6 neurons projecting to thalamus are spontaneously active and their activity increases during unspecific full-field visual stimulation (Figure 1D in Olsen et al., 2012). Surprisingly, when layer 6 activity is artificially increased in a highly correlated manner by broad optogenetic photostimulations or by full-field visual stimulation, it has a suppressive effect on the dLGN and on other cortical layers (Olsen et al., 2012). By contrast, silencing layer 6 via photostimulation of V1 inhibitory neurons strongly facilitates dLGN activity (Olsen et al., 2012). However, similar experiments in A1 and V1 had no visible effects on the average firing of auditory thalamic neurons (Li et al., 2013a), and of dLGN neurons (Li et al., 2013b).

Broad photostimulation and full-field stimulation may lack the specificity of physiological activity during natural perception and attention. In the optogenetic silencing experiments when the light is shone through the cortex, the spatially uniform activation and the temporal patterns generated in layer 6 GABAergic neurons and in inhibitory neurons in other layers may differ from that evoked by visual and auditory stimuli. The fact that the firing rate of thalamic neurons is often unchanged after silencing the cortex is likely a result of a concurrent decrease of excitatory drive from layer 6 and inhibitory drive from the TRN which also receives direct excitation from layer 6 of the cortex (Li et al., 2013b). When optogenetic photostimulation was more specific, targeting a layer 6 neuron sub-population, it was capable of driving both increases and decreases in visually evoked spike count, even in simultaneously recorded cells, without affecting burst frequency (Denman and Contreras, 2015). The observed effects result from a balance of monosynaptic excitation and disynaptic inhibition that can be tipped toward either inhibition or excitation.

These results point to the great complexity of the layer 6 circuits and are not contradictory with the proposed selective attention hypothesis. We show in particular that the decorrelation of top-down synaptic bombardment do not act on the average activity of individual thalamic relay cells. Therefore, it could be difficult to capture this effect with random multi-unit recording in the thalamus.

### Corticothalamic feedback and selective attention

The convergent synaptic organization of the thalamocortical circuit forms the structural kernel upon which selective attention may act. This topology of relay neurons in the visual system is essential because it provides a locus of control over the transfer of sensory information. First, it allows recipient cortical cells to average in a single trial the spiking probability function of each afferent relay cell, which is presumably controlled by the cortical input. This averaging distributed over the presynaptic thalamic population enables a reliable and rapid decoding of feedforward sensory signals. Second, thalamic output spike synchrony provides a means for feature selectivity in cortical area V1, turning cortical cells into synchrony detectors, and enabling the cortex to extract sensory features encoded by the coactivation of afferent relay cells. Modulations of the mean and variance of background synaptic noise may tune the transfer of sensory information by adjusting thalamic synchrony. Third, the concomitant synaptic bombardment exerted by the descending corticothalamic feedback results in a tunable stochastic facilitation of the feedforward inputs. This effect—controlled by synaptic noise decorrelation at the subthreshold level—, and possibly supervised by cortical areas, enables fine adjustments in transmitted sensory information, and therefore appears to be a potent mechanism by which the cortex may regulate the transfer of selected sensory signals during attention.

We suggested that oscillations, which are the hallmark of sleep and absence epilepsy, and are associated with a drop of sensory perception, could be another possible mechanism contributing to thalamic selective attention. The filtering of unattended sensory signals is possibly accounted by a default thalamic regime, characterized by weak thalamocortical ensemble oscillations, that leads to the synchronous entrainment of TC cells and results in a decoupling of sensory inputs from the cortex. By contrast, dynamic modulations exerted by the cortical feedback could provide a switch mechanism to augment the sensory throughput of the thalamus (Crandall et al., 2015), thus enabling a controlled transfer of sensory information to the cortex. We therefore propose that one important role of the cortical feedback is to decorrelate subthreshold synaptic noise and oscillations in order to adjust the transfer efficiency of selected sensory signals, informed by context and prior knowledge.

Our prediction is that background synaptic activity in thalamocortical regions focused by attention presents low correlations, while activity in neglected regions outside of the focus presents high correlations. Therefore, in agreement with the view of Foxe and Snyder (2011), we think that alpha/mu oscillations are not so much carrier waves for information propagation but rather the signature of information transfer suppression by providing uniformity to cellular responses. A prediction that remains to be tested is that activity correlations should be lower in thalamic and V1 regions that are selective to attended features. Experimental validation of this prediction remains a major issue because of the yet unsolved technical challenge of identifying and recording simultaneously all neurons belonging to a thalamic network that converges to the same recipient cortical cell. A challenging alternative would be to record simultaneously many TC cells intracellularly in order to unravel the correlation level of the synaptic bombardment during attentive tasks.

## Methods summary

### Ethics statement

All *in vitro* research procedures concerning the experimental animals and their care were performed in accordance with the local animal welfare committees and adhered to the American Physiological Society's Guiding Principles in the Care and Use of Animals, to Center for Interdisciplinary Research in Biology and European Guidelines Directive 2010/63/EU, to European Council Directive 86/609/EEC, to European Treaties Series 123, and were also approved by the regional ethics committee “Ile-de-France Sud” (Certificate 05-003). *In vitro* slice experiments were conducted in rodents bred in the Central CNRS Animal Care at Gif-sur-Yvette (French Agriculture Ministry Authorization: B91-272-105) under the required veterinary and National Ethical Committee supervision. Every precaution was taken to minimize stress and the number of animals used in each series of experiments. Animals were housed in standard 12 h light/dark cycles and food and water were available *ad libitum*.

### Slice preparation

*In vitro* experiments were performed on 300–350 μm-thick slices from the dLGN and VPm of Wistar rats 4–6 weeks old for sharp recordings, 14–25 days old for patch recordings, C57BL/6J wild-type or Cav3.1^−∕−^ (Kim et al., 2001) mice 14–23 days old, or adult guinea pigs. Animals were anesthetized with sodium pentobarbital (30 mg/kg) or inhaled isoflurane before decapitation, craniectomy and brain removal. The brain was rapidly removed and immersed in a cold “cutting” solution, and slices were prepared with a vibratome in which the NaCl was replaced with sucrose while maintaining an equivalent osmotic pressure. Slices were then incubated in a “recovery” solution for 30 min to 2 h before recording, in either interface style or submerged recording chambers.

### Electrophysiolgy

During recording, the slices were incubated in an oxygenated artificial aCSF. Whole-cell patch-clamp recordings and intracellular sharp recordings of TC neurons were performed using a Multiclamp 700B (Molecular Devices) or an AxoClamp 2B (Axon Instruments) amplifier. Dynamic-clamp was used to insert sensory-like inputs and cortically-induced synaptic noise in TC neurons.

### Dynamic-clamp

The dynamic-clamp technique (Robinson and Kawai, 1993; Sharp et al., 1993; Piwkowska et al., 2009) was used to inject computer-generated conductances in real neurons. When using sharp electrodes, dynamic-clamp was coupled with an Active Electrode Compensation (Brette, 2009) that allows the removal of electrode noise from intracellular voltage recordings in real time. The dynamic-clamp software is based on a custom ADC/DAC program used for data acquisition and analysis (Elphy2, developed at UNIC by Gérard Sadoc) and is interfaced with a real-time NEURON environment (Sadoc et al., 2009), in which the NEURON simulator v6.0 (Hines and Carnevale, 1997) was modified and recompiled to run under the INtime stack (TenAsys), a kernel driver enabling real-time operation under Microsoft Windows OS. Stimulation protocols were run in real time with the acquisition card at 10–20 kHz.

### Sensory input

Sensory glutamatergic AMPA inputs were mediated by conductance-based synaptic currents described by:

$$\begin{array}{l} I_{AMPA}=g_{AMPA}\left(E_{AMPA}-V_{m}\right) \end{array}$$

where *g*_*AMPA*_ is the evoked conductance in the post-synaptic compartment and *E*_*AMPA*_ = 0 mV the reversal potential.

### Synaptic noise

Background synaptic noise was simulated by fluctuating conductances generated as independent stochastic processes and mimicking the effect of thousands of stochastically glutamate- and GABA-releasing synapses (Destexhe et al., 2001). The total injected synaptic current *I*_*noise*_ was composed of two conductances, excitatory *G*_*exc*_ and inhibitory *G*_*inh*_:

$$\begin{array}{l} I_{noise}=G_{exc}\left(E_{exc}-V_{m}\right)+G_{inh}\left(E_{inh}-V_{m}\right) \end{array}$$

where *E*_*exc*_ = 0 mV and *E*_*inh*_ = −75 mV are the reversal potentials for the excitatory and inhibitory conductances, respectively.

### Data analysis

A spike was considered as a response to the input when it occurred within 20 ms after the stimulus onset. Responses were considered as multi-spike if the interspike interval was < 10 ms. The transfer efficiency of the retinocortical sensory signal transfer was calculated by means of mutual information theoretical analysis:

$$\begin{array}{l} MI\left(S;R\right)=\underset{s}{\sum }P\left(s\right)\underset{r}{\sum }P\left(r∕s\right){log}_{2}\frac{P\left(r∕s\right)}{P\left(r\right)} \end{array}$$

where *S* denotes the stimulation, *R* the response, *P*(*s*) the probability of presentation of the stimulus pattern *s*, *P*(*r*) the probability of presentation of the response pattern *r* and *P*(*r*∕*s*) the probability to obtain the response pattern *r* in response to the stimulus pattern *s*.

### Modeling and simulations

Model neurons were described with the equation:

$$\begin{array}{l} C_{m}\frac{{dV}_{m}}{dt}=I_{leak}+I_{Na}+I_{h}+I_{K}+I_{T}+I_{noise} \end{array}$$

where *V*_*m*_ is membrane potential, *C*_*m*_ is the capacitance of the cell, *I*_*leak*_ is the leak current, *I*_*Na*_ is the voltage-dependent Na^+^ current, *I*_*h*_ is the hyperpolarization-activated non-specific cationic current, *I*_*K*_ is the delayed rectifier K^+^ current, *I*_*T*_ is the T-type Ca^2+^ current, and *I*_*noise*_ is the synaptic noise.

The T-current was simulated by a Hodgkin–Huxley-like model:

$$\begin{array}{l} I_{T}=g\bar{C}am^{2}h\left(E_{Ca}-V_{m}\right) \end{array}$$

where $g\bar{C}a$ is the maximal conductance, and *m* and *h* the activation and inactivation variables, respectively. Activation and inactivation gates follow the simple two-state kinetic scheme introduced by Hodgkin and Huxley (1952).

Iteratively constructed biological networks were built using a two-step procedure. First, biological TC neurons were sequentially recorded under various stimulus conditions. Second, recorded membrane potentials were replayed off-line in a model circuit to simulate the synaptic convergence of the thalamic layer onto a model cortical cell.

### Conflict of interest statement

The authors declare that the research was conducted in the absence of any commercial or financial relationships that could be construed as a potential conflict of interest.

## Supplementary information

All recordings and analyses performed in this study are described elsewhere. For the ionic channel level, knock-out mice, TTA, and T-current protocols are described in Deleuze et al. (2012). For the neuron level, input-output transfer functions are described in Wolfart et al. (2005) and Deleuze et al. (2012). For the population level, biologically iteratively constructed networks, model circuits and mutual information transfer analysis are described in Béhuret et al. (2013).
